# FGF3 Directs the Pathfinding of Prethalamic GABAergic Axons

**DOI:** 10.3390/ijms241914998

**Published:** 2023-10-08

**Authors:** Hong Huang, Qingyi Chen, Zhengang Xu, Fang Liu

**Affiliations:** 1 Department of Cell Biology, School of Basic Medical Sciences, Nanchang University, Nanchang 330031, China; 2 Medical Experimental Teaching Center, School of Basic Medical Sciences, Nanchang University, Nanchang 330031, China

**Keywords:** FGF3, GABAergic axons, thalamus, axon guidance

## Abstract

The thalamus plays a crucial role in ensuring the faithful transfer of sensory information, except olfactory signals, to corresponding cortical areas. However, thalamic function is not simply restricted to relaying information to and from the cerebral cortex. The ability to modulate the flow of sensory information is supported by a second abundant neuronal type in the prethalamus, the inhibitory gamma-aminobutyric acid (GABAergic) neurons, which project inhibitory GABAergic axons to dorsal thalamic glutamatergic neurons. Interestingly, during the trajectory of pioneer prethalamic axons, morphogen fibroblast growth factor (FGF)-3 is expressed in the ventral chick hypothalamus. Using in vitro analyses in chick explants, we identify a chemorepellent effect of FGF3 on nearby prethalamic GABAergic axons. Furthermore, inhibition of FGF3 guidance functions indicates that FGF3 signaling is necessary to navigate prethalamic axons correctly. Gene expression analyses and loss of function studies demonstrate that FGF3 mediates prethalamic axonal guidance through the downstream pathway of the FGF receptor (FGFR)-1. Together, these results suggest that FGF3 expressed in the hypothalamus functions as a chemorepellent molecule to direct the pathway selection of neighboring GABAergic axons.

## 1. Introduction

The prethalamus consists of a sheet of gamma-aminobutyric acid GABAergic neurons, which are situated between the thalamus and the cortex. Prethalamic GABAergic neurons receive excitatory inputs from collaterals of thalamocortical and corticothalamic axons and provide the major inhibitory input to glutamatergic thalamic neurons [1,2]. Synaptic inhibition attenuates excitatory signals and prevents hyper-excitability of neurons, thus playing an essential role in the information processing of neural circuits. Previous studies have demonstrated that dysfunction of prethalamic GABAergic neurons is associated with diseases of the central nervous system, such as epilepsy, Alzheimer’s disease, and schizophrenia [3,4]. It is, therefore, crucial to understand the guidance mechanisms of early inhibitory prethalamic axons within the diencephalon. However, compared to the abundance of studies on the development of inhibitory circuits in the cortex, the maturation of thalamic inhibitory connections is poorly understood.

Fibroblast growth factors (FGFs) consist of 23 different polypeptide growth factors that communicate with FGF receptors (FGFRs) to play important roles in developing both vertebrates and invertebrates. Within the developing neural system, FGFs are reported to be required for patterning, neurogenesis, and maintaining the physiology and homeostasis of neurons [5,6]. In addition to these classical activities, FGFs are found, at later embryonic development, to function as axon guidance molecules at the level of cells [5,6]. For instance, FGF8, which mediates patterning activities of the midbrain–hindbrain boundary (MHB), repels axons of midbrain dopaminergic neurons (mDANs) extending through the diencephalon [7]. We previously also discovered that FGF3 from the hypothalamus guides the pathfinding of thalamocortical axons (TCAs) [8]. Interestingly, we found that during the growth of TCAs, prethalamic axons also start extending into the thalamus. Thus, we explored the effects of hypothalamic FGF3 on the development of prethalamic axonal projections. Here, from in vitro analyses of diencephalic explants and in vivo expression assays, our studies demonstrate an indispensable chemorepulsive effect of hypothalamic-derived FGF3 on the trajectory of prethalamic GABAergic axons using the model of chick embryos. In chick, FGF3 binds two transmembrane FGF receptors (FGFR1 and FGFR2) [9]. Our studies indicate that only FGFR1 is significantly co-expressed by prethalamic GABAergic cells. Moreover, downregulated FGFR1 function revealed that FGFR1 signaling is the main downstream regulator of FGF3-mediated prethalamic axon guidance. These data support our hypothesis that FGF3 plays a direct role in the navigation of prethalamic GABAergic axons and suggest a mechanism by which FGFR1 signaling is involved in the pathfinding of prethalamic axons.

## 2. Results

### 2.1. Inhibitory GABAergic Axons in the Developing Chick Prethalamus

**Figure 1:**
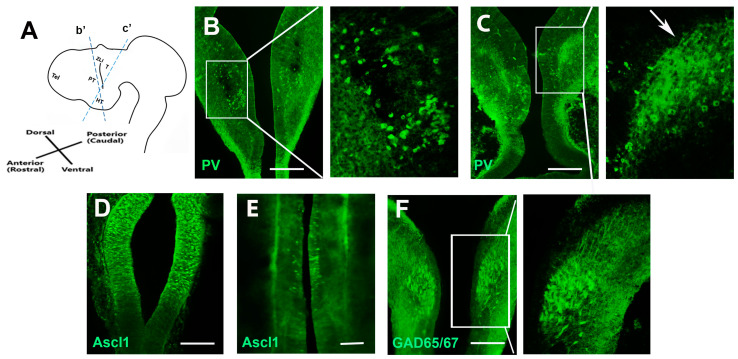
Expressions of prethalamic marker genes. (**A**) Schematic showing a lateral view of the forebrain. (**B**) A section of an E5 embryo at the level of the prethalamus (indicated in (**A**)—b’) shows PV+ neurons are found throughout the prethalamus. The right-hand panel shows a high-power view of the boxed region. (**C**) A section through the prethalamus and the thalamus (indicated in (**A**)—c′) shows PV+ prethalamic axons (arrow) extending towards the thalamus at E5. The right-hand panel shows a high-power view of the boxed region. (**D**) The GABAergic progenitor marker Ascl1 is widely detected in the E3 chick prethalamus. (**E**) Sporadic expression of Ascl1 is restricted in the ventricular zone of the E5 prethalamus. (**F**) GAD65/67 immunohistochemistry expression in the prethalamus at E5. T, thalamus; PT, prethalamus; HT, hypothalamus; Mb, midbrain; ZLI, zona limitans intrathalamica; Tel, telencephalon. Scale bars represent 100 µm.

**Figure 2:**
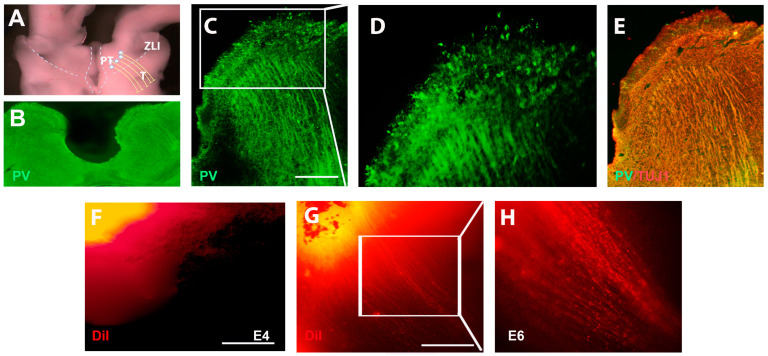
Early prethalamic axonal trajectory in the thalamus. (**A**) The model shows a prethalamic trajectory in the section of the ‘open-book’ diencephalon. (**B**) Transverse section of the ‘open-book’ diencephalon at E6. (**C**) Immunohistochemical analyses of PV on the ‘open-book’ diencephalon. (**D**) High-powered view of boxed region in C. PV+ neurons project axons towards the thalamus. (**E**) Co-analysis of PV and TUJ1 on the section of the thalamus. (**F**) DiI labeling reveals that no obvious prethalamic axons extend towards the thalamus at E4. (**G**) By contrast, many DiI-labeled prethalamic axons extend towards the thalamus at E6. (**H**) Shows a high-powered view of the boxed region in G. Scale bars represent 100 µm.

Previous studies have shown that the calcium-binding protein parvalbumin (PV) is widely distributed in prethalamic GABAergic neuronal cell bodies, dendrites, and axonal fibers [10]. Similar to the expression in the mouse, at the prethalamic section level (Figure 1A—b’), the GABAergic subtype marker PV is detected throughout the prethalamus (Figure 1B). At E5, a section was taken along the prethalamus–thalamus axis and tested for the expression of PV on prethalamic axons (Figure 1A—c’). Our studies show that PV+ pioneering axons project into the posterior thalamus at E5 (Figure 1C). Previous studies indicated that the basic helix–loop–helix (bHLH) transcription factor Ascl1 (=Mash1) was shown to be expressed only in progenitor GABAergic cells [2]. Consistent with these studies, abundant Ascl1 positive progenitor neurons were observed at E3 in the prethalamus (Figure 1D). In contrast, at E5, only a few Ascl1-positive cells are found in the ventricular zone of the prethalamus (Figure 1E). Similar to the expression of PV, the pan-markers of GABAergic inhibitory neurons GAD65/67 genes are widely detected in the chick prethalamus at E5 (Figure 1F). Most descriptions of prethalamic GABAergic axons are based on postnatal descriptions, but there are few studies of developing prethalamic axons. To identify the pathway of developing prethalamic axons, ‘open-book’ diencephalon explants (Figure 2A) were dissected out and sectioned (Figure 2B) at E6, and then PV and axonal marker TUJ1 (anti-neuron-specific class IIIβ-tubulin) expressions were detected using immunohistochemistry. PV-positive prethalamic neurons project GABAergic axons into the thalamus, forming ordered and parallel projections (Figure 2C–E). To further track the trajectory of early prethalamic axons in the thalamus, we used DiI-tracing experiments on ‘open-book’ diencephalic explants. The lipophilic carbocyanine dye, DiI, diffuses anterogradely or retrogradely specifically along neuronal processes and cell bodies. Anterograde DiI labeling shows the parallel trajectory of growing GABAergic axons protruding dorsally to the thalamus at E6 but not E4 (Figure 2F–H).

At E5, when pioneer prethalamic GABAergic axons are projecting towards the thalamus, a number of FGF3 expressions are expressed in the hypothalamus of chick embryos [8]. Thus, the patterns of FGF3 expression in the hypothalamus, together with the pathway selection of PV+ prethalamic axonal bundles, suggest that prethalamic axons grow selectively away from the FGF3+ hypothalamus and enter towards the thalamus.

### 2.2. FGF3 from the Hypothalamus Directs Prethalamic Axons

**Figure 3:**
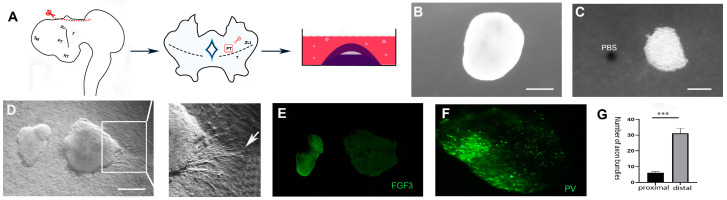
FGF3+ hypothalamus directs prethalamic axons in chick. (**A**) After isolating the ectodermic forebrain at E4, a cut is made above the diencephalon. Then, according to experimental requirements, different parts of the diencephalon will be dissected and then cultured in collagen for two days. (**B**) Few axonal outgrowths are examined in prethalamic explants cultured alone. (**C**) Minimal thalamic axons grow out from prethalamic explants that are cultured with PBS beads. (**D**) Axons extend out from prethalamic explants co-cultured with FGF3+ hypothalamic explants, and these prethalamic axons appear to be repelled by hypothalamic explants (arrow). (**E**) FGF3 expression in the hypothalamic explant. (**F**) PV+ neurons in the prethalamic explant. (**G**) Quantitative analysis of axon bundles from proximal versus distal faces of prethalamic explants (*n* = 6 explants). Error bars represent s.e.m., ***: *p* < 0.001. Scale bars represent 100 µm.

**Figure 4:**
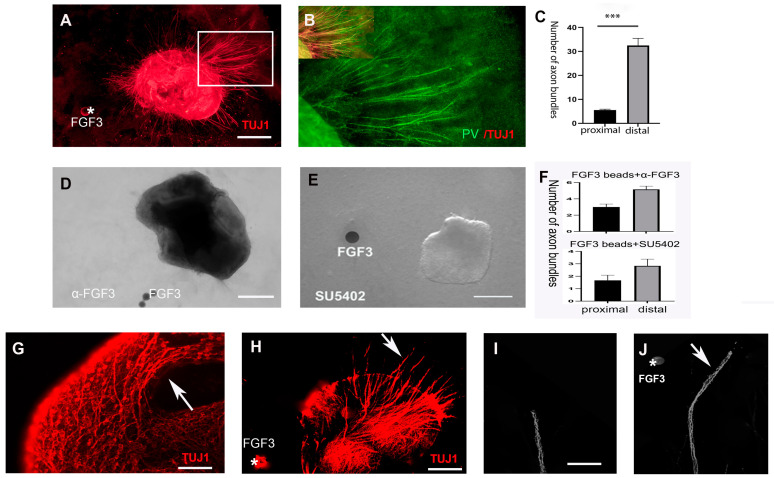
FGF3 repels prethalamic axons in the thalamus. (**A**) In the presence of FGF3 beads (asterisk), TUJ1+ prethalamic axons grow extensively and mainly accumulate on the distal side. (**B**) Co-analysis of PV and TUJ1 on prethalamic axons of box area in H shows that among TUJ1+ axons, some PV axons are repelled by FGF3-soaked beads (300 ng/mL). (**C**) Statistical analyses from summed data from *n* = 8 explants reveal a significant difference between the number of axon bundles projecting away and towards the FGF3 beads. (**D**) The axonal-repellent effect of FGF3 is reduced in the presence of an FGF3-blocking antibody. (**E**) With FGFR inhibitor SU5402 in the culture media (20 µM), axon responsiveness to the chemorepellent FGF3 beads is abolished. (**F**) The difference in axon outgrowth from separate sides is not significant in prethalamic explants grown with FGF3 beads in the presence of an FGF3 blocking antibody or SU5402 (*p* > 0.05) (*n* = 7 explants). (**G**) Immunolabeling shows prethalamic axons (arrow) circling around the peripheral component when there are no guidance cues. (**H**) Immunolabeling on frozen sections shows that axons (arrow) project out from prethalamic explants in the presence of FGF3 (asterisk). (**I**) An axon bundle extends from a 24 h cultured prethalamic explant. (**J**) In the presence of an FGF3 gradient (asterisk) for four hours, the axon makes a turn (arrow) in the opposite direction to FGF3. Error bars represent s.e.m., ***: *p* < 0.001. Scale bars represent 100 µm in (**A**,**D**,**E**); 50 µm in G and H; 25 µm in (**I**,**J**).

To test whether FGF3 is required to navigate developing prethalamic axons, the E4 diencephalon was isolated from surrounding tissues. After Dispase treatment, distinct diencephalon sections were dissected and then cultured in collagen for 2 days (Figure 3A). When prethalamic explants are cultured alone or with phosphate-buffered saline (PBS) beads, few prethalamic axons are observed in the collagen (Figure 3B,C). Previous studies have shown that, without chemotropic cues in the environment, axons choose to circle around the peripheral area of the explants instead of projecting outside [8]. However, when PV+ prethalamic explants are co-cultured with FGF3+ hypothalamic explants, hypothalamic explants induce the outgrowth of prethalamic axons, but these axons are repelled and grew oppositely from the hypothalamic source (Figure 3D–F). Quantitative analyses reveal a significant difference in the number of axons projecting from different sides of prethalamic explants, with significantly more prethalamic axons in the distal section (away from FGF3 beads). (Figure 3G). To test whether FGF3 might be the repellant molecule mimicking the effects of the FGF3+ hypothalamus, prethalamic explants were then cultured with FGF3-soaked beads in collagen gels. In comparison to prethalamic neurons grown alone, FGF3 beads (300 ng/mL) can significantly promote PV+ axonal outgrowth and repel these axons away from the FGF3 sources (Figure 4A and B). Quantitatively, the number of prethalamic axons is significantly greater in the distal section (away from FGF3 beads) than in the proximal section (towards FGF3 beads) (Figure 4C). To address the specific effects of FGF3, we use FGF3-blocking antibodies on co-cultures of FGF3 beads and prethalamic explants. In vitro, inhibition of FGF3 induces a marked decrease in axon outgrowth evoked by FGF3 beads (Figure 4D). To determine whether FGFR signaling is involved in the guidance effects of prethalamic GABAergic axons, explants were exposed to FGFR inhibitor SU5402. With SU5402 in the media, FGF3 beads fail to direct prethalamic GABAergic trajectory; few prethalamic axons are examined in the collagen gel (Figure 4E). Unlike FGF3, SU5402 and FGF3-blocking antibodies did not induce a significant difference in the number of proximal and distal axons (Figure 4F). Consistent with the above statement, without guidance cues around, instead of extending out of the explants, prethalamic axons circle around the peripheral area of the explants (Figure 4G). However, with FGF3 beads, prethalamic axons project out of gels in the opposite direction to FGF3 sources using frozen sections (Figure 4H). To further examine the mechanisms of prethalamic guidance effects of FGF3, we carried out turning experiments. Firstly, prethalamic explants were cultured in a normal culture medium for 24 h (Figure 4I). Then, 300 ng/mL FGF3 beads were added to produce the gradients. As depicted in Figure 4H, a repulsive response was strongly observed within 4 h (Figure 4J).

Together, these data suggest that FGF3 is one of the guidance molecules released from the developing hypothalamus, directing the accurate navigation of early prethalamic axons.

### 2.3. FGF3 Is Required in the Correct Targeting of Prethalamic Axons

**Figure 5:**
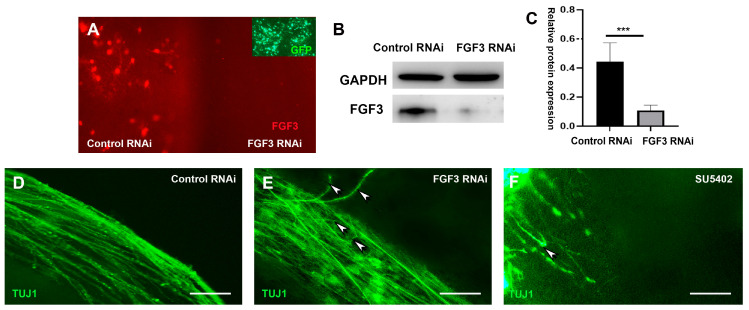
FGF3 is required for the navigation of prethalamic axons. (**A**) Chick diencephalon explants cultured in whole-mount view, with one side targeted with FGF3-RNAi vectors. The other half side acts as a negative control with the electroporation of control vectors. Inset: GFP expressions on both sides. (**B**) Western blot analyses of FGF3 levels reveal a decrease in FGF3 levels after FGF3 RNAi electroporation compared to the control side. (**C**) Quantification of (**A**) shows summed data from *n* = 6 explants. ***: *p* < 0.001 versus the controls. (**D**) Electroporation of negative control vectors shows prethalamic pioneer axons project towards the thalamus. (**E**) FGF3 knockdown induces disordered axons, with some axons turning in an opposite direction (arrowheads). (**F**) With the addition of SU5402, both the number and the length of the axons are decreased, accompanied by incorrect axonal turning (arrowhead). Scale bars represent 50 µm.

To further address whether FGF3 exerts a direct guidance effect on pioneer prethalamic axons, we performed RNA interference (RNAi) to downregulate FGF3 gene expression. In contrast to the negative control of the no-specific electroporated side, the FGF3 ShRNA expression system works evidently, achieving downregulated expressions of FGF3 in the developing hypothalamus (Figure 5A). There was a significant decrease of FGF3 expressions in the electroporated side compared with the no-specific electroporated side using analyses of Western blotting (Figure 5B,C).

To avoid confusion with outer lateral TCAs, diencephalic explants were sectioned, and the intermediate prethalamic axons were exposed. Compared to negative controls with non-specific ShRNA expression (Figure 5D), electroporation of the FGF3-RNAi vector induces disordered axonal growth. Instead of entering the thalamus, some prethalamic axons turn around and grow in the opposite direction (Figure 5E). Additionally, with FGFR inhibitor SU5402 in the culture media, our data show that prethalamic axonal extension is significantly reduced in the thalamic explant, and just a few short and misdirected axonal fascicles can be examined (Figure 5F).

### 2.4. Roles of FGFR1 Signaling in the Guidance of Prethalamic Axons

**Figure 6:**
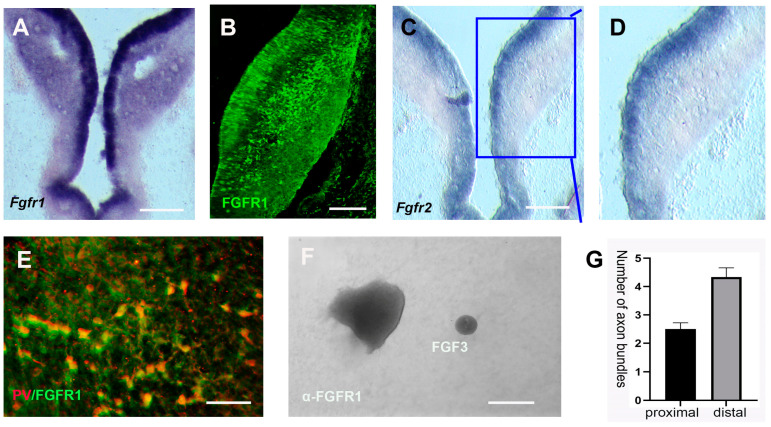
FGFR1 signaling is required for the correct trajectory of prethalamic axons. (**A**–**D**) FGFR1 and FGFR2 mRNA, as well as FGFR1 protein expressions in transverse sections. (**E**) Double immunofluorescence of FGFR1 and prethalamic GABAergic marker PV in prethalamic cells. (**F**,**G**) When cultured with an FGFR1-blocking antibody, few axons are detected. The number of prethalamic axons on the proximal side (toward FGF3 beads) does not statistically differ from axons on the distal side (away from FGF3 beads; *p* > 0.05, *n* = 6 explants). Scale bars: 100 μm in A, C; 50 μm in (**B**,**E**,**F**).

To determine the downstream receptor of FGF3, expressions of FGF3 receptors (FGFR1 and FGFR2) were analyzed. Our results show that FGFR1, but not FGFR2, is widely expressed in the developing prethalamus at E5 (Figure 6A,D). We performed a co-immunofluorescence assay to understand the molecular mechanism underlying the FGF3 activation in the prethalamic axonal navigation. At E5, PV+ GABAergic neurons in the prethalamus are co-expressed with FGFR1 (Figure 6E). To address the specific requirements for FGFR1, we analyzed the effects of FGFR1-blocking antibodies on co-cultures of FGF3 beads and prethalamic explants. In the absence of FGFR1 signaling, there is a substantial reduction in the number of axons repelled by FGF3 beads (Figure 6F). Furthermore, there is no significant difference in the number of axons protruding from the proximal and distal sides (Figure 6G). These results suggest that FGFR1 signaling is necessary for the chemorepulsive effect of FGF3 on the pathfinding of prethalamic GABAergic axons.

## 3. Discussion

Establishing the correct neural circuit requires precise navigation of developing axons, which depends upon a complex system of guidance molecular cues within the environment. Growth cones at the end of growing axons are guided by a combination of positive (chemoattractive) and negative (chemorepellent) cues, which may function as substratum-tethered short-range cues or via long-range diffusible cues [11]. FGFs, a large family of secreted polypeptides, interact with FGF receptors (FGFRs) to control various developmental processes at early stages, such as cell division, proliferation, differentiation, survival, migration, etc. However, at later developmental stages, FGFs play a significant role in establishing the nervous system’s complicated neuronal networks [12,13].

Normally, growth factors are considered chemoattractive molecules short of manipulating cyclic nucleotides [14,15]. However, FGFs have been proven to act as chemorepulsive molecules on developing axons. For example, in vivo axons of retinal ganglion cells (RGC) avoid FGF-misexpressing cells along their path, and in vitro RGC growth cones are directly repelled by FGF-2 in a growth cone turning assay [16]. Nevertheless, in contrast to the huge number of studies on the chemoattractant effects of FGFs, the chemorepellent effects of FGFs are poorly understood. Here, we investigate the axon guidance effects of FGF3 during the trajectory of prethalamic GABAergic axons. Our studies indicate that FGF3 exerts strikingly direct repellent effects on prethalamic axons in chick explant cultures. Moreover, analyses of downregulated FGF3 and FGFR1 signaling demonstrate that the indispensable repellent effects of hypothalamic FGF3 on developing prethalamic axons are mediated through the FGFR1 downstream signaling pathway.

### 3.1. Direct Guidance Effects of FGF3 on Developing Prethalamic Axons

FGF3 expression is initially detected in the ventral hypothalamus [17]. However, at E5, consistent with previous studies [17], FGF3 expression spreads to the dorsal hypothalamus during the pioneer trajectory of prethalamic axons [8]. In the diencephalon, several FGFs, such as FGF15/19, FGF8, FGF10, and FGF3, are expressed [8,17,18]. Among these FGFs, FGF3, from the FGF7 family, has been well reported as an important guidance factor in the diencephalon. FGF signaling maintains a subset of ventral progenitor cells in a proliferative, undifferentiated state in the hypothalamus at early development stages [19]. At later development stages, FGF3 elongates the outgrowth of hypothalamic axons and directs distinct hypothalamic neurosecretory axons to their specific targets [17]. Prethalamic inhibitory GABAergic neurons, which are anterior to the zona limitans intrathalamica (ZLI) line, will give rise to the reticular nucleus of the thalamus (TRN). These prethalamic GABAergic neurons (TRN GABAergic neurons) are marked by a GABAergic subtype marker PV (parvalbumin), which is a calcium-binding protein expressed in most TRN neurons [20]. Our studies reveal that at E5, specific prethalamic TRN marker PV and panGABAergic marker GAD65/67 are widely detected in the chick prethalamus [2]. Thus, considering the adjacent expressions between hypothalamic FGF3 and prethalamic PV during the navigation of prethalamic GABAergic axons, we continue to explore the guidance effects of hypothalamic FGF3 on the pathfinding of inhibitory prethalamic fibers. In rodents, most TRN neurons are generated in the prethalamus at E13–15 [21]. DiI-labeled parallel TRN axons are observed in dorsal relay nuclei at E14 [22]. Our studies extend these observations, showing that some PV+ axons appear to extend into the thalamus at chick E5. However, at E4, no obvious PV+ prethalamic axons were found in the thalamus. Consistent with previous studies [21,23], anterograde DiI labeling and immunolabelling show that parallel prethalamic GABAergic axons protrude dorsally into the thalamus. Previous studies indicate that prethalamic pioneer axons somehow help the passage of the TCAs across this region [23]. Our studies also demonstrate that during the navigation of TCAs in the prethalamus, PV+ prethalamic fibers start extending to the thalamus. Previous studies also show that, in transverse sections, TCAs project ventrally along the lateral surface of the ventral thalamus [24,25]. However, fibers of prethalamic axons appear in the intermediate regions of the thalamus. Thus, our research shows that TCAs and prethalamic GABAergic axons do not appear at the same level in the diencephalic cortex, which may explain why TCAs can guide prethalamic axons without interfering with each other.

To avoid complicated in vivo interference, we use a well-recognized in vitro technique, the 3D (three-dimensional) collagen co-culture method, to identify the putative chemorepulsive properties of FGF3. Our previous studies indicate that when there are no guidance cues in the environment, most axons will not grow outside the explants; however, when there are guiding molecules in the environment, instead of circling around the edge of explants, axons will grow out of the explants showing attraction or rejection [8]. Firstly, prethalamic axons are found to extend out of the explants and turn away from the FGF3 source directly in vitro, suggesting that FGF-dependent chemorepulsion is one of the explanations for the trajectory of prethalamic GABAergic axons which avoid FGF3+ hypothalamus in vivo. Moreover, analyses of downregulated FGF3 signaling demonstrate an indispensable guidance role of hypothalamic FGF3 in developing prethalamic axons. Within the diencephalon, FGF3 acts synergistically with FGF8 to govern the patterning of the developing ventral thalamus at relatively early embryonic stages [26,27]. Previous studies indicate that the patterning function of FGFs begins at very early stages of development, and the remaining FGFs play a significant role in establishing the complicated neuronal network of the nervous system [8,17]. Consistent with these studies, our studies add evidence to these theories that the reorientation of axons via hypothalamic FGF3 at later stages is distinct from its patterning effects. Studies show that thalamic progenitor GABAergic cells are marked by the expression of bHLH transcription factor Ascl1 [2]. Our results also demonstrate that at E5, the number of Ascl1+ progenitor cells is significantly less than progenitor cells at early stages, Such as E3. These results suggest that the guidance effects of FGF3 in the pathfinding of prethalamic axons are unlikely to have corresponding consequences of compromised differentiation and development of prethalamic neurons. In sum, these data strongly suggest a necessary chemorepulsive effect of FGF3 on the navigation of prethalamic axons.

### 3.2. FGF Signaling Is Required in the Correct Targeting of Prethalamic GABAergic Inhibitory Axons

SU5402 interacts with the catalytic domain of the FGFRs, thereby suppressing the tyrosine kinase activity of all FGF receptors [28]. Our data reveal that the addition of SU5402 disrupts the chemotropic activity of hypothalamic FGF3. These results indicate that FGFR signaling supports the chemorepellent effect of FGF3 in guiding prethalamic GABAergic axons toward the thalamus. Although SU5402 is widely used as an inhibitor of FGFR signaling, it does not distinguish whether it is FGFR1 or FGFR2 signaling that contributes to the prethalamic chemorepulsive effects of FGF3.

The progenitor region of the diencephalon is subdivided into three transverse domains along the AP (anterior–posterior) axis: prosomere 1 (p1), prosomere 2 (p2), and prosomere 3 (p3). The pretectum, thalamus, and prethalamus are derived from the alar plate of p1, p2, and p3, respectively [29]. In mice, FGFR1 exhibits expression only in p3, whereas both FGFR2 and FGFR3 exhibit a gradient of expression in p2 and p3 of the thalamus [27]. This also suggests the importance of FGF activity in developing diencephalon. Consistent with these studies, among two FGFRs of FGF3, only FGFR1 is widely expressed in the prethalamus at E5 chick. Moreover, FGFR1 and PV were co-expressed in prethalamic GABAergic cells. Among FGFR1-4, FGFR1 is the most abundant fibroblast growth factor receptor in the nervous system and is primarily expressed by neurons [30]. We thus propose that the FGF3 chemorepulsion effect on prethalamic axons is mediated through the FGFR1 downstream pathway in the developing thalamus. We found that downregulated FGFR1 on prethalamic explants blocks FGF3-mediated repulsion of prethalamic axons. Moreover, the prethalamic axonal abnormality caused by SU5402 and FGFR1 inhibitor addition is much more severe than that caused by an FGF3-blocking antibody alone, which not only suggests the necessity of FGFR signaling in the projection of prethalamic axons but also indicates other FGFs may play a role in the pathfinding of prethalamic axons, such as FGF10 in the hypothalamus.

Thus, these results provide support that FGF3 signaling works through the FGFR1 downstream pathway to repel prethalamic GABAergic axons in the thalamus. Several transduction pathways can be activated via FGFR1 signaling, such as the RAS-mitogen-activated protein kinase (RAS-MAPK), phosphatidylinositol 3-kinase (PI3K)-serine/threonine kinase (AKT), phospholipase C gamma (PLC*γ*), and non-canonical phosphatidylcholine-specific phospholipase C (PC-PLC) intracellular signaling pathways [5,16,31]. In the future, it will be important to investigate the roles of other downstream components of FGFR1 signaling in the pathfinding of prethalamic GABAergic axons. In summary, this study is the first presentation to show that hypothalamic FGF3 affects the trajectories of prethalamic pioneer axons extending to the thalamus by using a well-established 3Dculture system and indicates that FGF3 can act as a chemorepellent molecule mediated by a FGFR1 signaling pathway within the thalamus.

## 4. Materials and Methods

### 4.1. Explant Culture

Local brown chicks from the Jiangxi Breeding Factory were used. All embryos were staged and Dispase isolated (1 mg/mL, Basel, Swiss, Roche). Then, the diencephalic neurectoderm was dissected in an ‘open-book’ presentation (Figure 2A). According to the requirements of distinct experiments, different sections of the hypothalamus and thalamus were dissected. Explants were then cultured in collagen beds (344010001, Minneapolis, MN, USA, R&D) based on published techniques [32]. When two explants were cultured together, the distance between them was around 100–300 µm. Affigel blue gel beads (153-7302, Hercules, CA, USA, BioRad) were washed three times in phosphate-buffered saline (PBS) and then soaked in FGF3 protein (R&D) overnight at 4 °C prior to culture. The FGF3 protein was standard at 300 ng/mL; its antagonist SU5402 (Calbiochem, Darmstadt, Germany) was used at 20 µM.

### 4.2. Immunofluorescence Analyses

Analyses of embryos and explants were analyzed using immunohistochemistry according to standard whole-mount or cryostat sectioning techniques [33]. In this study, antibodies used were: anti-TUJ1 (1:1000 dilution; ab 78078, Abcam, Cambridge, UK); anti-FGF3 (produced by ABclonal, Wuhan, China; 1:200); anti-GAD65/67 (1:1000 dilution; ab11070, Abcam, Cambridge, UK); anti-parvalbumin (1:200 dilution; PV, bs-1299R, Bioss, Beijing, China); anti-FGFR1 (1:200 dilution; bs-0230R, Bioss, Beijing, China); and anti-green fluorescent protein (GFP) mouse monoclonal (ab1218, Abcam, Cambridge, Uk). Secondary antibodies were conjugated to Alexa 647 or Alexa 488 (1:1000 dilution; Jackson, Lancaster, PA, USA); FGFR1 antibody blocking peptide (bs-0230P, Bioss; 50 ng/μL); and FGF3 antibody blocking peptide (bs-1255P, Beijing, China, Bioss; 50 ng/μL).

### 4.3. DiI Tracing

DiI labeling experiments were performed on E6 (embryonic day 6) ‘open-book’ thalamic explants. The lipophilic carbocyanine dye, 1, 1-dioctadecyl-3, 3, 3′, 3′-tetramethyl-indocarbocyanine perchlorate (DiI) (KeyGEN BioTECH, Nanjing, China) was injected into the 4% paraformaldehyde (PFA) fixed ventral prethalamus (anterograde experiments). The explants were fixed in 4% PFA for a month and then examined.

### 4.4. RNAi Electroporation

RNAi targeting vectors designed by Shanghai Genechem (Shanghai, China) express a short hairpin RNA (ShRNA) directed against the FGF3 sequence GCTTGTTCTCTGGCAGATA from a microRNA operon expression cassette (U6-MCS-CMV-GFP-SV40-Neomycin). The FGF3-RNAi vector was electroporated into the E4 whole-mount embryonic diencephalon. A non-specific RNAi vector (against TTCTCCGAACGTGTCACGT), which has no homology to any known chicken gene sequences, was taken as a negative control. The electroporation procedure was as previously described [34]; the maximum DNA concentration in all electroporations was 0.5 μg/μL. After 48 h of incubation, primary analysis was performed by directly observing the location and morphology of GFP+ cells. Then, the expression patterns of TUJ1/PV were analyzed for defects caused by RNAi targeting of FGF3 (*n* = 6).

### 4.5. In Situ Hybridization

Embryos were processed for in situ hybridization as described previously [33]. Following development, embryos were analyzed on cryostat sections.

### 4.6. Western Blot Analysis

After dissecting the diencephalon, FGF3-RNAi was electroporated into one side of the E4 diencephalon, and the other side was used as a control. After 48 h of incubation, the FGF3-RNAi electroporated and control sides were separated equally. The explants were cut into 3 mm × 3 mm small pieces and then lysed in a radio immunoprecipitation assay (RIPA) lysis buffer containing complete mini protease-inhibitor cocktail tablets (Roche, Mannheim, Germany). The total protein concentration in the supernatants was estimated using MD Spectramac M3, US. A Western blot analysis was performed as previously described [35]. The following antibody dilutions were used: anti-glyceraldehyde-3-phosphate dehydrogenase (GAPDH) (KeyGen, Nanjing, China; 1:2000) and anti-FGF3 (ABclonal, Wuhan, China; 1:500). The intensity of the bands was quantified using Gel-Pr32 software.

### 4.7. Statistical Analysis

Statistical analyses were carried out using SPSS22.0 for PC and graphed using GraphPad Prism 9.0 software. The statistical significance of differences in means between axons on distinct sides was analyzed using an independent *t*-test. *p*-values less than or equal to 0.05 indicate a significant difference. All experiments were repeated at least six times.

## Data Availability

Data will be made available upon request.
